# The impact of poverty and deprivation at the end of life: a critical review

**DOI:** 10.1177/26323524211033873

**Published:** 2021-09-12

**Authors:** Jane Rowley, Naomi Richards, Emma Carduff, Merryn Gott

**Affiliations:** End of Life Studies Group, School of Interdisciplinary Studies, University of Glasgow, Glasgow, UK; End of Life Studies Group, School of Interdisciplinary Studies, University of Glasgow, Glasgow, UK; Marie Curie Hospice Glasgow, Glasgow, UK; Professor, Te Ārai Palliative Care and End of Life Research Group, School of Nursing, The University of Auckland, Private Bag 92019, Auckland 1142, New Zealand

**Keywords:** deprivation, end of life, inequalities, inequity, palliative care, poverty, social determinants of health, social gradient

## Abstract

This critical review interrogates what we know about how poverty and deprivation impact people at the end of life and what more we need to uncover. While we know that people in economically resource-rich countries who experience poverty and deprivation over the life course are likely to die younger, with increased co-morbidities, palliative care researchers are beginning to establish a full picture of the disproportionate impact of poverty on how, when and where we die. This is something the Covid-19 pandemic has further illustrated. Our article uses a critical social science lens to investigate an eclectic range of literature addressing health inequities and is focused on poverty and deprivation at the end of life. Our aim was to see if we could shed new light on the myriad ways in which experiences of poverty shape the end of people’s lives. We start by exploring the definitions and language of poverty while acknowledging the multiple intersecting identities that produce privilege. We then discuss poverty and deprivation as a context for the nature of palliative care need and overall end-of-life circumstances. In particular, we explore: total pain; choice at the end of life; access to palliative care; and family caregiving. Overall, we argue that in addressing the effects of poverty and deprivation on end-of-life experiences, there is a need to recognise not just socio-economic injustice but also cultural and symbolic injustice. Too often, a deficit-based approach is adopted which both ‘Others’ those living with poverty and renders invisible the strategies and resilience they develop to support themselves, their families and communities. We conclude with some recommendations for future research, highlighting in particular the need to amplify the voices of people with lived experience of poverty regarding palliative and end-of-life care.

## Introduction

Death exposes in high relief the layers of inequities, in race and income, care and opportunity, that shape life down to its final hours. It is a truth the pandemic has only underscored — one hard to see, because it is so much easier to look away.^1^

Covid-19 has brought inequities in end-of-life circumstances sharply into focus. In particular, the disproportionate impact of the pandemic on people experiencing poverty has made it clear that social factors have a major influence upon when, why and how people die. There is an urgent need to address these inequities as part of international postpandemic recovery efforts,^2^ including within the context of palliative care.^3^

In this article, we interrogate the available research to see if it is sufficient to address this agenda and inform equity-focused practice and policy improvements in palliative care. Our focus is particularly upon deprivation and poverty, terms we recognise as value laden and contested. Indeed, we begin our discussion by interrogating the language used in this field in more detail, identifying challenges for researchers in achieving conceptual clarity, as well as acknowledging the stigma and shame which particular terminology can engender.

We adopt a critical social science lens to examining the literature. We align ourselves with Stajduhar^4^ in recognising that privilege is key to securing access to palliative care, and that privilege is as invisible as it is powerful. Moreover, although in this article we focus on socio-economic differentials in accessing palliative care, we understand that privilege is produced through multiple intersecting identities. We also recognise that the nature and organisation of palliative care, including the research that we ourselves do, is shaped by the aspirations of majority populations. The imagined palliative care patient around which policy continues to be formulated lives in secure housing, with family support and is white, middle class and male.^5^ While starting from a sound knowledge of existing literature, we chose not to undertake a systematic or scoping review because we wanted to bring an eclectic range of sources together from both within and beyond palliative care to see if we could shed new light on the myriad ways in which experiences of poverty shape the end of people’s lives. We were particularly interested in identifying gaps in current knowledge to shape a research agenda moving forward. As such, this discussion complements the growing number of reviews in this area focused on discrete aspects of this topic, for example, Davies and colleagues^6^ and Lewis and colleagues^7^ by taking a broader ‘big picture’ perspective.

Our discussion ranges from: the impact of poverty upon palliative care need; the attenuation of choice at end of life for those with limited means; access to and utilisation of generalist and specialist palliative care; and family caregiving. On the rare occasions where evidence permits, we foreground the voices of people with lived experience of navigating their end of life within the context of poverty, although this is one of the most notable gaps we identified in the current literature.

Overall, we recognise that in addressing poverty and deprivation, there is a need to recognise not just socio-economic injustice, but also cultural and symbolic injustice.^8,9^ Too often, a deficit-based approach is adopted which both ‘Others’ those living with poverty^10^ and renders invisible the strategies and resilience they develop to support themselves and their families and communities.^11^ As Fogarty and colleagues^11^ argue, deficit discourses ‘narrowly situate responsibility for problems with the affected individuals or communities, overlooking the larger socio-economic structures in which they are embedded’. This framing is evident in language used to justify the under-representation of people with lived experience of poverty in research. Denoting such groups as ‘hard to reach’ focuses attention upon the research participants themselves, rather than the paucity of research where researchers are sufficiently embedded in the community to build trusting relationships.^12^ We consider this in our conclusion which, congruent with our intention to progress the field through prompting critical reflection and discussion, includes concrete recommendations for a future research and policy agenda.

This discussion paper is confined to the context of economically resource-rich countries because meanings and experiences of poverty are very different in economically resource-poor countries. Moreover, palliative care is either at a very early stage of development or nonexistent meaning that the challenges faced also differ. However, we note an urgent need to attend to palliative care development in resource-poor countries, not only to support improved end-of-life care, but also so mutual learning can occur as has happened, for example, in public health palliative care.^13^ Finally, where there is a range of evidence to draw from, we provide examples from Scotland and Aotearoa New Zealand as these are the countries in which we live and work.

## Definitions

*What is Poverty? Who asks? Who answers?* ^14^

The first thing to say is that the very definition of poverty is value laden. The reason for this is that *how* it is defined determines how it is measured, which determines its extent, and ultimately, determines the pressure that comes to bear on politicians to do something about it. This Lister calls the ‘moral imperative’ of poverty.^15^ The longest running conceptual debate among poverty researchers is between ‘absolute’ and ‘relative’ definitions. Absolute poverty is understood as not being able to meet the bare minimum – subsistence level–necessary for human survival.^16^ This relates to long-standing attempts to draw a ‘poverty line’ in monetary terms, below which people can be said to be experiencing poverty. Relative poverty, on the contrary, is deemed to be when a person is unable to obtain the ‘diets, amenities, standards and services’ which are customary, or ‘widely encouraged or approved of’ in the society in which they live.^17^

There are two researchers we find particularly instructive in escaping the ‘rather sterile’ debate between these two positions.^8^ Both Lister^8^ and Spicker^18^ argue for a multi-dimensional view of poverty that recognises a material core to poverty whereby the person experiences unacceptable hardship, but that around this core there are other dimensions. Lister^8^ identifies relational-symbolic dimensions which give rise to a sense of diminished citizenship and powerlessness, alongside experiences of being stigmatised. Spicker^18^ identifies three ‘clusters of meaning’ around the term poverty: material need; people’s economic circumstances; and people’s social position. For both researchers, poverty is not just about inequality: it is about lacking certain basic things over an extended period of time, resulting in a low standard of living and an inability to flourish.

The word ‘deprivation’ is often used interchangeably with the word poverty within the literature. Deprivation can be understood to denote a lack of the things that are needed, whether that is lack of food, clothing, fuel, shelter, (material deprivation) or lack of social connection (social deprivation). People are essentially said to be ‘deprived’ when they lack something they need. Poverty is broader than deprivation because, if we take the multi-dimensional view, it incorporates people’s economic circumstances and social position as well. According to Spicker,^18^ people can be deprived without being poor, because lacking a specific need might amount to something less than poverty. However, he states that there are not many senses of poverty which do not involve deprivation in some form. For this reason, throughout this discussion paper, we use the inclusive phrase ‘poverty and deprivation’.

Given that the concept of poverty is contested, ways of measuring poverty are also contested and therefore vary. There is no universally agreed upon or ideal measure. We can measure people’s income, income inequality, and area deprivation levels, but these are ‘indicators’ and not the same as measuring poverty in all its multi-dimensional complexity.^18^ Depending on the topic under investigation and given known associations between area deprivation and health service availability and accessibility, an area-based approach can be useful, as explored further below. However, there are clearly limitations to using geographical data alone, most notably that living in a ‘deprived area’ does not mean that you are necessarily experiencing poverty. This is particularly the case when researching rural poverty where households are more spread out and even more heterogeneous.^19,20^

In this article, we draw on literature which has used a range of approaches to measure deprivation and poverty, including income and socio-economic status. Where literature is limited, we also draw on work which has looked at related indicators such as educational level and social class, although recognise that there are clearly inherent difficulties in simply equating these measures with poverty and deprivation. A critical lens is also required because, as Fu and colleagues^21^ recognise, deprivation measures used without ‘critical reflection can lead to deficit framing of populations and maintain current inequities in health and social outcomes’.

Indeed, language matters and in considering definitions it is important to remember that it is people with the greatest power in society who determine how ‘Others’ are described. As Lister argues even the word ‘poor’, despite its purportedly innocent economic descriptive, carries stigmatising power^8^ because the term is used by the non-poor to define and label people in poverty without ever taking into account people’s own self-definition.^8,10^ Indeed, a key gap in the poverty literature, and one which must be addressed by palliative care researchers as a foundation to developing new research programmes in this area, is to explore with people experiencing deprivation how they think language should be used for research purposes. In this vein, we return to the work of Chambers, whose words started this section, and who argues:

If we are seriously pro-poor professionals, the answer to “What is poverty?” is “That is the wrong question”. It is our question, not theirs. The questions are then: whose reality counts? Ours? Or theirs? Or more precisely: ours, as we construct it with our mindsets and for our purposes? Or theirs as we enable them to analyse and express it?^14^

## Impact of lifetime poverty and health inequities on ‘total pain’ at the end of life

The poorest people have high levels of illness and premature mortality – but poor health is not confined to those who are worst off. At all levels of income, health and illness follow a social gradient: the lower the socioeconomic position, the worse the health. (WHO Commission on Social Determinants of Health, & World Health Organization. (2008)).

There is now widespread acceptance that the social conditions in which people are born, grow, live, work and age have a significant influence upon their health across the life course. The World Health Organisation^22^ has identified 10 inter-related social determinants of health. The social gradient, as it is called, indicates that the higher a person’s socio-economic position, the better their health. This association starts in childhood, with the poorest experiencing the highest rates of infant mortality and the wealthiest the lowest rates,^22^ and continues into adulthood. People who experience poverty across the life course are more likely to die younger, and even in high-income countries,^23^ there can be a 20-year gap in life expectancy between the most- and least-deprived areas.^24,25^ The number of years someone can expect to live in good health also follows a social gradient. In Scotland, for example, the gap in healthy life expectancy at birth between the poorest and richest areas is 25.1 years for men and 21.5 years for women.^26^ The incidence,^27^ course and outcome of most chronic diseases follows the social gradient^28^ and associations extend into later life, including for both cognitive function and frailty.^29^ The increased likelihood of complex co-morbidities across the life course is reflected in primary care workloads, with Scottish general practices in the most deprived decile experiencing 38% more patients with multi-morbidity compared with the least deprived.^30^ There is also evidence that people with fewer years of education experience poorer physical health even in the last months and years of life likely reducing their quality of life as they approach death.^31^ People of lower socio-economic status have also been found to experience a shorter time period between diagnosis of a life-limiting illness and death.^27^

While this suggests an association between poverty and more complex palliative care needs, research in this area is limited. A recent UK qualitative study interrogating the concept of complexity did, however, recognise that this could be impacted by lifetime social circumstances.^32^ As one participant noted: ‘forget about the complexity of [the] illness, the complexity of just normal life is much higher’^32^ (p. 1082). In this study, Cicely Saunders’ concept of ‘total pain’ was invoked by the authors to frame people’s complex needs at the end of life across many domains. Total pain is a concept which tries to encapsulate how pain experienced at the end of life is a whole overwhelming experience which has physical but also psychological, social and spiritual elements.^33^ While difficulties have been identified with the concept, notably in terms of an expansion of the ‘medical dominion’ into new areas of human suffering,^34^ it provides a useful starting point for considering how the social determinants of health impact on people’s end-of-life experiences.

The ‘social’ dimension of total pain is the most neglected within the literature and relates to the interpersonal connections between the dying individual and her social world.^33^ It provides a useful way in to considering the ways in which ‘pain and distress can be produced by the social: by inequality, marginalization, injustice, powerlessness and persecution’.^35^ There is certainly evidence that, at end-of-life, current and lifetime experiences of social inequities can compound suffering.^35,36^ As Finucane and colleagues^37^ argue, social needs relate to factors such as social isolation, caring responsibilities, housing concerns, or family/carer support needs, discussed further towards the end of this article.

There is also evidence that poverty has an impact on physical pain, with significant associations identified between socio-economic disadvantage and experiences of pain across a range of pain sites.^38,39^ For example, an Austrian study^40^ found that socio-economic status, measured through education, income, and profession ‘was inversely and gradually associated with the prevalence of severe pain, with the number of indicated painful body sites, the intensity of pain, and with the subjective level of feeling disabled through pain’. A social gradient in pain experience was reported whereby, at the same intensity of pain and number of painful body sites, people in the lowest socio-economic group were two to three times more likely to report feeling disabled through pain. Self-reported pain has also been found to be significantly related to subjective household income among US cancer patients, although the authors note the moderating influence of educational level.^41^

Implications for palliative care are unknown, although this evidence indicates that people experiencing poverty and deprivation are more likely to experience pain prior to the onset of a life-limiting condition, which is then likely to shape how pain is experienced at end of life. Previous experiences of seeking treatment for pain will also play a role and there is evidence that people experiencing poverty can struggle to access sufficient pain medication due to health professional prejudice regarding drug-seeking behaviour,^42^ including in a palliative care context.^43^ Health professionals have also been found to be more responsive to pain experienced by affluent patients.^44^ This evidence points to a picture whereby people experiencing deprivation may be more likely to experience pain at the end of life, but less likely to receive appropriate pain relief. However, more research is needed and an intersectional lens recommended because of known associations between other social identities and experiences of pain, for example, gender.^5^ Indeed, previous research confirms that: ‘intersecting sociodemographic factors create unique social identities that impact pain’.^39^

Similar associations have been found between poverty and deprivation and other commonly experienced end-of-life symptoms, all of which can cause people to suffer. For example, in a US study, Muni and colleagues^45^ found that people with lower income dying in the intensive care unit (ICU) had higher levels of dyspnea when they were assessed by clinicians,^45^ although overall received fewer assessments. That rates of dyspnoea are higher among people experiencing poverty and deprivation is not surprising given considerable evidence indicating that lung function is negatively correlated with socio-economic status across the life course, even when controlling for smoking status, occupational exposures and ethnicity.^46^ The influence of sub-standard housing is a likely contributory factor.^47^ Research also indicates that the experience, and response to, dyspnea is shaped by social context and a model of ‘total dyspnea’, similar to that of ‘total pain’, has been proposed.^48^ How social context may impact symptom experience is exemplified by findings from a study in a former mining town with high smoking rates–Barnsley, England–where chronic obstructive pulmonary disease (COPD) was viewed as a normal part of ageing. In particular, people with COPD and their families rarely sought treatment because,^49^ as one study participant put it, in that town ‘you get old, you get breathless, and you die’. This indicates that how end-of-life symptoms are interpreted and if, and how, they are discussed with health professionals is shaped by the socio-cultural context within which people live and have unique aspects for people living in areas of deprivation. However, more research is needed to fully understand implications for clinical practice.

Psychological and emotional well-being represents a further dimension of total pain. There is strong evidence of an association between area deprivation and lower mental health throughout the life course,^50^ including into older age.^29^ A social gradient has also been identified earlier in the life course, with research establishing a link between childhood poverty and poor mental health in adulthood.^51–54^ Importantly, this association holds both for those who continue to experience poverty, and for those who experience improved economic circumstances in later life.^55^ However, there is also evidence of a social gradient in terms of access to mental health services. For example, UK GPs working in areas of deprivation have reported reluctance in identifying and treating patients with depression because they regard the wider structural and social factors underpinning their mental health problems as insoluble.^56^

What this means regarding the experience of mental health problems for people living with deprivation and poverty specifically at the end of life remains unclear, although there is some evidence of an association between lower socio-economic status and increased anxiety and depression among patients receiving palliative care.^48,57^ However, again more research is needed.

The final, and another neglected,^42^ dimension of Saunders’ concept of total pain is spirituality and previous research indicates that the role it plays within the context of poverty requires further investigation. For example, a study in Aotearoa New Zealand^58^ examining one expression of spirituality, namely religiosity, found that people living in deprived neighbourhoods reported higher subjective wellbeing than their non-religious counterparts living in the same areas. The protective effects of spiritually derived systems of meaning to help people cope with adversity has been discussed widely, although the negative effect on health of certain belief systems must also be acknowledged.^59^ A need for future research in the area of spirituality to attend to social diversity, including the context of poverty and homelessness, has been identified.^60,61^

Interestingly, in an early article by Balfour Mount,^62^ an instrumental figure in establishing the specialism of palliative care in Canada, financial pain was added as another dimension of ‘total pain’:

Saunders has coined the term ‘total pain’ to describe the all-consuming nature of chronic pain and our need to attack all of its components – physical, psychological, financial, interpersonal and spiritual.^62^

For the early pioneers of palliative care then, financial worries and struggles were part and parcel of the whole overwhelming experience of total pain and were acknowledged to cause or exacerbate general suffering at the end of someone’s life. This is particularly the case in privatised healthcare systems where uninsured individuals can fall through the cracks and struggle to find ways of paying for their care and treatment. It is our view that there is scope for theoretical exploration of the conceptual overlaps between the concepts of total pain, complexity and the social determinants of health.

## End of life ‘choices’

What are the ethical implications of asking people to make choices about their preferences for end of life care if our formal care systems lack capacity and our social networks are inadequate to fulfil these preferences? (Grindrod, 2020)^63^

Neoliberal health policies of the 21st century^64^ present choice as a way to solve the problem of dying.^65^ Indeed, in palliative care policy, practice and research, providing choice has become an indicator of good end-of-life care in and of itself.^66^ However, as Borgstrom and Walter argue: ‘Informed choice, whether at the end of life or in advance of it, does not guarantee the death the person wants, especially for those dying of conditions other than cancer’,^66^ (pg 1). We would extend this argument to include people experiencing poverty and deprivation. There is no evidence that solely offering choice translates into improved patient outcomes for this population, or even that choice is desired. Indeed, if by the nature of their life circumstances people experiencing poverty have had limited opportunity to make choices about where they live, how they generate income and what they eat, how do they begin to make choices about the end of their lives? In her work Passage,^67^ a body of photographic work which explores inequality throughout the life course, Mitchell speaks of the nature of choice:

I want the viewer to ask themselves a question about how society operates, how choice is related to opportunity and environment. To see that sometimes people choose what they do, because really, not much has been offered in the first place. (Mitchell, 2020)^67^

The patient as consumer framework is also predicated on an individualistic decision-making model which is not congruent with the collective systems of belief of many non-western and Indigenous cultures.^68^ Moreover, it also presupposes that everybody has the same social and economic resources available to draw on to formulate and enact choices. However, this is clearly not the case. A good example can be found with regards to a decision point universally prioritised in palliative care discourse, namely place of care and, in particular, place of death.^69,70^ Palliative care policy internationally prioritises dying at home^71^ and many countries have directed efforts to increasing home deaths and reducing those that occur in institutions, particularly acute hospitals.^72^ This has meant that palliative care access in some countries is becoming more focused on home settings, limiting access opportunities for those people experiencing poverty who are vulnerably housed.^73^ The imagined home in a policy context is also warm and comfortable: it is spacious enough to accommodate hospital equipment and health professional visitors, there is food in the fridge and family on hand to provide care and support.^74^ What if this is not the case? In Aotearoa, New Zealand, almost one in five people live in sub-standard housing,^75^ and approximately 10% of the population struggle to access sufficient food, with indigenous Ma¯ori most at risk.^76^ In England and Wales,^77^ there are nearly 30,000 excess winter deaths, attributed to cold weather and high energy prices. Rates of homelessness are rising in many resource-rich countries particularly among older people;^78^ in Australia for example, rates of homelessness among women over 55 increased 31% between 2011 and 2016.^79^ There is limited understanding of what dying ‘at home’ means in this context.^80^ As one older man living in the North of England reported in a qualitative study exploring end-of-life decision-making: my home is not a nice place to live, what makes you think it would be a nice place to die?^81^

However, for some the desire to die ‘at home’ may override any concerns about poor material circumstances, but choosing to die in a setting, or in a way which may not be congruent with wider societal understandings of a ‘good death’ can lead to tension. For example, Kellehear^82^ argues that, for older people,

Dying alone is viewed either as an outcome of anti-social behaviour or the result of family, neighbourhood or social services neglect. The idea that people may be exercising agency, resistance or dissent at the end of life and that they do not want attention from services, or the wider community receives little or no consideration.

This argument can be extended to people living in deprivation, with previous research identifying that clinicians make moral judgements about a patient’s circumstances which can impact approaches to treatment.^83–86^ Here, navigating autonomy and risk can be challenging, something not acknowledged within the patient as consumer framework, which envisages that people will be able to make choices about their care that will ‘favour themselves and promote their own lives’.^87^ It also assumes people will make choices which align with normative understandings of good ways to die.

This is interesting to consider when reflecting on research which has identified that people experiencing poverty and deprivation appear to receive more ‘aggressive’ end-of-life treatment, defined within the research literature as an increased intensity of interventions such as ICU admissions, resuscitation, ventilator support, use of Emergency Department (ED) and non-palliative chemotherapy.^88,89^ These types of medical intervention have been used as proxy markers of lower quality of death.^90^ However, this is not a universally held view. Minoritized populations – notably black Americans – consistently express preferences for these types of end-of-life intervention.^91^ Reasons for this, in particular a lifetime of experiencing inequities in access to services,^92^ including healthcare,^93^ might also apply to those experiencing poverty and deprivation. However, more research is needed to explore the congruence between end-of-life preferences, treatment received and quality of end-of-life care from the patient and family perspective.

Another explanation for this pattern of end-of-life treatment may be that people experiencing poverty are less likely to engage in Advance Care Planning and/or complete Advance Directives than those who are more affluent. For example, in a US study, Barwise and colleagues^94^ identified a social gradient in uptake of Advance Directives among adults in the Intensive Care Unit, with the lowest uptake among those in the lowest socio-economic group. The authors speculate that this finding may be related to higher rates of education and health literacy among people of higher socio-economic status as associations have been reported between lower levels of education and health literacy and reduced likelihood of engaging in Advance Care Planning conversations.^94,95^ Indeed, while evidence of an association between poverty and Advance Care Planning is limited, Canadian ethnographic research has identified that immediate concerns about survival trumped long-term concerns such as advance care planning for people experiencing structural vulnerability. As one physician participant put it, people are just so ‘busy living in the moment and surviving’.^73^

## Use of palliative care services within the context of poverty and deprivation

Even though palliative care as a concept seems to be unanimously supported, that is what it remains: an idea that only becomes reality for people privileged enough to access it. Those who do benefit tend to also benefit from high socio-economic status and family support. People who are facing the end-of-life who also face social and structural inequities like poverty, homelessness, racism, and stigma, are not so privileged.^4^

In most economically resource-rich countries, universal palliative care provision is envisaged at a policy level as being facilitated through a specialist/generalist model.^96^ Specialist palliative care should be provided to those with the most complex needs; generalist palliative care to the remainder through existing health providers such as GP and hospital-based teams. Our discussions to this point indicate that people experiencing poverty deprivation and are likely to have more complex palliative care needs. If access to palliative care services is equitable, we would therefore expect this population to be *more* likely to receive palliative care from both specialist and generalist providers. However, the limited evidence available points, in the main, to the opposite being true.

A recent systematic review provides a good overview of available evidence. Davies and colleagues^6^ explored the

association between socio-economic position (e.g., income, education, occupation, private medical insurance status, housing tenure, housing quality, or area-based deprivation) and place of death, plus use of acute care, specialist and non-specialist end-of-life care, advance care planning, and quality of care in the last year of life.^6^

A total of 209 relevant studies were identified, 53.5% from North America, 31.0% from Europe, 8.5% from Australia, and 7.0% from Asia. Findings demonstrated that people living in the most deprived neighbourhoods were less likely to receive specialist palliative care and more likely to experience an acute hospital admission in the last 3 months of life and to die in hospital. This finding is also supported by studies published more recently; for example, Buck and colleagues^97^ research reports that people from lower socio-economic groups in England are less likely to receive hospice at home than those who are more affluent. Davies and colleagues^6^ conclude that: ‘Low socioeconomic position is a risk factor for hospital death as well as other indicators of potentially poor-quality end-of-life care, with evidence of a dose response indicating that inequality persists across the social stratum’.

However, the nature of palliative care received in hospital, and whether hospital death is viewed as a ‘poor quality’ end-of-life outcome for people with lived experience of poverty and deprivation, requires further reflection as evidence in the literature is mixed. Survey-based data from Aotearoa New Zealand, for example, concluded that people with palliative care needs living in areas of deprivation report more benefit from hospital admission than those living in more affluent areas.^98^ The study was not designed to explain this association, but data from other countries outside of a palliative care context provide some insights. For example, US research confirms that people experiencing deprivation and associated challenges of family dysfunction, mental health concerns and homelessness viewed the hospital as a safe space where they can connect with other people.^99^ As one study participant reported: ‘In the hospital it was quiet. Come home, it’s chaos’.

However, conversely, a Canadian ethnography identified that for some of their structurally vulnerable participants: ‘hospitals symbolized the inflexible and oppressive systems of institutional control’.^100^ Trauma related to colonialism was recognised as a particular context for Indigenous people, who often avoided seeking treatment for pain until it became unbearable, which was often close to death. These divergent data remind us of the need to adopt an intersectional lens when considering the social context of end-of-life experience.

Barriers to receipt of palliative care identified in previous research have not yet been explained, although Lewis and colleagues^7^ hypothesise that they are related to the availability, affordability, acceptability and geographical accessibility of palliative care.^101,102^ A key finding was that the ‘supply or availability of a service is not sufficient for access’. Literature from the US in particular confirmed feelings of stigma and distrust inhibited engagement with palliative care, although little research has explored this in other contexts. Indeed overall, the authors concluded that ‘Knowledge of access to palliative care services for low socioeconomic populations is limited’.

A separate body of work has identified specific challenges in delivering palliative care to people who are vulnerably housed and homelessness^103^ and illuminated their heightened experience of stigma in mainstream settings. Problematic use of drugs including alcohol have well-evidenced correlations to poverty^104^ and homelessness^105^ and a need to further develop our understanding of palliative care in this area has been identified.^106,107^ Intravenous drug use can reduce life expectancy by over 30 years^108^ and alcohol misuse impacts at least 28% of palliative care patients.^109^ Palliative pain relief for people in addiction is contentious.^43^

The nature of palliative care input to meet the needs of people experiencing poverty and deprivation is unknown, although there is evidence from the service provider side that more resource may be required to achieve, what they view as, the same level of care. For example, a study in London found that twice as many homecare visits were required by palliative care nurses in areas of deprivation to achieve similar rates of home deaths as those in affluent areas.^110^

In many resource-rich countries, primary care remains a key provider of generalist palliative care.^111^ With this setting there is strong evidence that the ‘Inverse Care Law’ operates. First proposed by GP Julian Tudor-Hart^112^ in a 1971 paper this ‘Law’ proposes that ‘The availability of good medical care tends to vary inversely with the need for it in the population served’.^112^ There is strong evidence that it continues to operate in the present day. For example, a study of over 3,000 Scottish patients concluded that there is an

increased burden of ill health and multimorbidity in poor communities result[ing] in high demands on clinical encounters in primary care. Poorer access, less time, higher GP stress, and lower patient enablement are some of the ways that the inverse care law continues to operate within the NHS and confounds attempts to narrow health inequalities.^113^

In countries where primary care services are not free at the point of access, this pattern is even more pronounced.^114^

There is some, albeit limited, evidence that this picture influences palliative and end-of-life care provision. For example, UK research has identified that primary care teams provide less out of hours palliative care in areas of high deprivation than more affluent areas. The authors of this study postulate that the likely reason for this is the higher burden of ill-health and associated primary care workload within these communities leads to less time to deliver palliative care.^115^ Areas of economic deprivation can also struggle to attract health professionals and GPs in such practices are at higher risk of burnout.^116^ While there is a lot of work being done by general practices to find novel ways to support their patients, communities and staff,^117^ GPs and community nurses often find their work in these areas challenging,^118^ particularly because of the barriers they experience in accessing support to address the social problems their patients experience.^119^ Additional support and training for primary care clinicians providing palliative care in these areas has been identified.^32^

It is also important to remember that people experiencing poverty and deprivation may already have contact with services and potentially hold trusted relationships with individuals who, with the requisite knowledge, have the potential to support palliative and end-of-life care. This extends traditional understandings of generalist palliative care and points to a need for reconceptualising the nature of palliative care provision for people experiencing poverty and deprivation. A good example is the Canadian Equity in Palliative Approaches to Care (ePAC) collaborative which involves ‘people with lived expertise, researchers, clinicians (such as doctors, nurses and counsellors), inner city workers, chosen family members and friends, administrators, and volunteers who work together to break down the silos in which structurally vulnerable people fall^65^’. This model of working focuses on strengths rather than deficits to engender and support pre-existing and organically emergent ‘compassionate communities^120^’ for populations experiencing poverty, housing vulnerability, and other associated challenges. Underpinned by public health principles, such an approach also recognises the inherent danger of palliative care in medicalising social aspects of people’s lives – their experiences of ‘total pain’ – and that many of the needs and concerns people have at end of life are not medical, but social. The very nature of ‘palliative care’ in this context therefore requires further consideration.

## Family caregiving

Sometimes … the children are fed, and we adults just have the leftovers, so we can make ends meet. But .. we always think of it’s temporary, and whenever the day will come, it will end. (Tongan daughter providing end of life care in Aotearoa New Zealand)^121^

Covid-19 has exacerbated existing trends in many economically resource rich countries regarding an increase in the amount, and complexity, of support and care undertaken by the family and friends of people with palliative care needs,^122^ particularly women.^123^ Indeed, while health policies aimed at reducing hospital use and increasing home dying at the end of life are presented as ‘what most people want’, the associated shift of financial costs from statutory services onto family and friends has been identified as a key equity issue for palliative care.^124^ The economic implications of increased responsibility for care are particularly profound for those already struggling to make ends meet. However, most literature has not addressed the added complexities of end-of-life caregiving within the context of poverty and deprivation.

That family will devote significant time and energy to supporting the person with palliative care needs ‘no matter what the cost’ was identified in a study exploring the economic costs of caregiving conducted in Aotearoa, New Zealand.^121^ However, the cost for those already struggling financially – many of whom were Indigenous Ma¯ori or Pacific people – was significant. Caregivers went without food themselves, incurred significant credit card debt and declared bankruptcy to ensure their family member had what they needed to be comfortable at the end of their life. In line with what we know about the gendered nature of end-of-life caring,^5,71^ women disproportionately shouldered caring responsibilities and were particularly at risk of long-term negative economic disadvantage resulting from stopping work or missing out on educational opportunities.^125^

Evidence regarding the availability of family support for people living in poverty is mixed. While there is a common perception that family members may be more likely to live close together and be ‘tight knit’, particularly within white working-class neighbourhoods, there is some evidence that people experiencing poverty may have access to less family support, not more. Matthews and Besemer^126^ challenge the ‘folk narrative’ conception of people living in deprived areas helping each other in times of crisis; while people may access short-term assistance from family and neighbours, this does not offer a reliable approach to the care someone may require at the end of life. The shame of poverty can also lead to people disengaging from communities and potential sources of support.^127^ In addition, the need to continue in paid employment may reduce the capacity of family members to be available to undertake caring tasks.

Previous research^128^ has identified that family caregivers of lower socio-economic status are more likely to experience moderate to severe depression when caring for someone with palliative care needs. This might be expected, given evidence discussed previously in this article regarding the association between deprivation and poorer lifetime mental health, as well as the additional complexities of caring within the context of poverty. It indicates more support may be required by family caregivers providing palliative care and experiencing poverty, although the nature of this (aside from a clear need for additional financial support) remains unclear. There is also evidence that people experiencing deprivation and poverty may have unique needs following bereavement. Drawing on critical social theory and the concept of disenfranchised grief, a recent scoping review^129^ identified four studies which addressed the impact of poverty and deprivation on bereavement. There was some evidence that heightened grief and increased vulnerability following bereavement was related to deprivation and socio-economic status and the authors concluded that:

the cumulative weight and dominance of stressors associated with financial and practical matters appeared to contribute to varied emotional and psychological difficulties.^129^

Policies which aim to support family carers of people with palliative care needs may have the unfortunate effect of compounding inequities by being blind to the needs of workers in precarious employment situations. The Canadian Compassionate Care Benefit, for example, is a 6-week government-funded scheme which provides family members with up to 55% of their usual income when caring for someone with a prognosis of <26 weeks,^130^ but part time, seasonal workers, and people who are unemployed are not eligible. The complexity of welfare systems can be a barrier to families accessing benefits they are entitled to when caring for someone at the end of life and those experiencing poverty and deprivation may find the system particularly complex to navigate.^131^ Moreover, many people who provide care and support for family at end of life do not identify as a ‘carer’,^132^ which represents a barrier to accessing financial support which is available and the system typically does not work quickly enough to provide urgent financial support such as that needed when someone is diagnosed with a life-limiting illness.^133^ Finally, what constitutes ‘family’ within this context and thereby who is eligible for any of the support which *is* on offer is also a key consideration. For example, Giesbrecht and colleagues^130^ found that vulnerably housed and homeless people considered their family to be their ‘street family and friends’, but were not seen as such by the legal and healthcare system.^65,134^ Overall, there are considerable gaps in our understanding of how experiences of poverty intersect with other structural vulnerabilities and how these experiences impact on people at the end of life. There is much to be done in investigating and understanding both the impact of poverty and the needs of people experiencing poverty at the end of life, as well as those that support them.

## Recommendations

In writing this article, we wished to identify whether the current evidence-base is sufficient to address an agenda of equity-focused practice and policy improvements in palliative care, with a particular focus on poverty and deprivation. It is clear that this is far from the case. We have identified significant gaps in current knowledge and understanding. While we are aware of,^135^ and involved with,^136,137^ projects designed to address these gaps, as a discipline we have a long way to go.

Most notably, we know very little from the perspective of people dying within the context of poverty and deprivation. Rectifying this absence must be a priority. As Beresford and colleagues argued over 20 years ago, the inclusion of people with lived experience in poverty discussions ‘is part of the broader issue of addressing the restricted citizenship of people who are poor. It also signifies respect…and that their contribution is important, worthwhile and valued’ (p. 27).^138^ In the absence of their voices, preferences and experiences driving and shaping the future research agenda, the risk of ‘Othering’ and deficit framing remains high. Moreover, building relationships between people with lived experience and researchers is fundamental to addressing the distrust in health research many structurally vulnerable populations experience.^139^ Strengths-based participatory approaches will be critical to such relationship building and lessons can be learned from research frameworks developed by Indigenous people which attend to issues of power, representation and self-determination.^140^ We also recognise the potential of public health palliative care approaches which are sensitive to the social determinants of end-of-life circumstances,^63^ particularly in supporting community-driven initiatives. Finally, an intersectional^141^ approach is important as we do not live ‘single issue lives’.^142^ Experiences of social forces such as colonialism, racism, sexism, homophobia and ableism compound the effects of poverty and deprivation.

Below we make some explicit recommendations, with the caveat that this list is by no means exhaustive but rather serves as a starting point for further discussion.

### Study design and research methods

- Ensure *all* research related to end-of-life care considers poverty and deprivation^6^ alongside the development of focused research programmes in this area;
- Develop a common language for researching palliative care in the context of poverty and deprivation in collaboration with people with lived experience of poverty;
- Understand the most appropriate measures to identify people – and regions – experiencing poverty and deprivation within a palliative care context;
- Explore the ways in which health systems can routinely capture data about patients experiencing deprivation and poverty in a non-stigmatising manner so as to better understand the impact of deprivation upon health and healthcare utilisation;
- Promote conversations with people and communities experiencing deprivation to better understand appropriate and acceptable research methods, with a particular focus on the potential benefits of using community-based participatory action,^143^ ethnographic,^73^ creative and visual methods;^136^ Include people with lived experience throughout the research process, from study design to delivering outcomes.

### Palliative care needs and preferences

- Understand how the context of poverty and deprivation shapes experiences of pain and other physical and psychological symptoms at end of life, along with their treatment;
- Explore the meaning and role of spirituality for people with a life-limiting illness experiencing poverty and deprivation and ways spiritual care can be optimised;
- Examine the utility of Saunders’ concept of ‘total pain’ to bring about a more holistic understanding of the multi-dimensional nature of suffering at the end of life;
- Explore the conceptual overlap between Saunders’ concept of ‘total pain’ and the social determinants of dying;
- Better understand the end-of-life choices of people experiencing poverty and deprivation and how these can be realised;
- Examine attitudes towards Advance Care Planning for people experiencing poverty and deprivation and mechanisms to support conversations about end-of-life preferences where these are desired;
- Explore preferences for the involvement of family (however this is defined) at the end of life;
- Further understand the associations between access to, and satisfaction with, specialist and generalist palliative care for people experiencing poverty and deprivation;
- Explore the perceptions of palliative care among populations experiencing deprivation and potential issues relating to trust and stigma;
- Identify the extra support needed by health and social care professionals to support equitable high-quality palliative care for people experiencing poverty, and/or living in areas of deprivation, including additional financial resources and education/training;
- Examine the role of institutional settings such as acute hospitals and Aged Residential Care facilities in supporting people who are experiencing poverty and deprivation at end of life – what experiences or innovations can they share?
- Explore the meaning of home for people living in precarious housing or who are homeless within the context of understanding preferred settings of care and death.

### Family caregiving

- Better understand the nature of the support that would help families that are providing care at the end of life, and following bereavement, within the context of poverty and deprivation;
- Identify policy and practice changes required to ensure financial support for family caregivers who need it most and share best practice internationally;
- Outline ways to promote interagency working to help caregivers to navigate the help and support which is available;
- Further understand the role played by family in providing end-of-life care within the context of poverty and deprivation, especially when applying an intersectional lens;
- Increase the representation of people who have lived experience of poverty and deprivation in the health and social care sector, particularly in fields in which they are traditionally under-represented, notably medicine; in the United Kingdom, only 4% of doctors come from lower socio-economic backgrounds backgrounds;^144^
- Embed research and knowledge about the social determinants of death and dying into undergraduate and postgraduate medical, nursing and social work programmes, using approaches which have been shown to work.^145,146^

### Increase clinical teaching about the social determinants of end-of-life experience to inform practice

- Increase clinical placements for students in areas experiencing deprivation, especially in general practice;^147^
- Increase understanding about how shame and stigma operate in the context of poverty and deprivation;^148^
- Explore the additional resource requirements of general practices and other health services located in, or serving, populations experiencing deprivation;
- Understand better the role of other (non-health-related) sectors in promoting good end-of-life care, for example, agencies with responsibility for housing and how to best connect them with health services.

### Research to inform policy

- Mainstream work on the social determinants of health into palliative and end-of-life care strategies and other policies relevant to this area;
- Identify mechanisms to promote policy integration of health, social care and other agencies which currently support, or have the potential to support, people experiencing deprivation and poverty;
- Establish good practice in taking a participatory approach to policy-making in palliative care which will ensure good practice in line with the Copenhagen Declaration and Programme of Action at the UN World Summit for Social Development which states that

People living in poverty and their organisations should be empowered by involving them fully in the setting of targets, and in the design, implementation, monitoring and assessment of national strategies and programmes for poverty eradication and community-based development.^149^

## Conclusion

The Covid-19 pandemic has made visible the impact of deprivation and poverty on people living with serious advanced illness at the end of life. We hope this will translate into an increased focus on, and resourcing for, research and policies which tackle some of the priorities we have identified above. However, significant change will be required.

Indeed, as the epidemiologist and prominent health equity researcher, Michael Marmot, stresses – instead of ‘build back better’, as the current political slogan goes, countries need to ‘build back fairer.^150^’ This will require greater redistribution of public funds to those who need them most. For those of us who work in the palliative and end-of-life care field, it will mean that we need to engage with wider policy agendas, beyond specialist palliative care, and beyond healthcare systems alone. Finally, for the agenda to truly move towards equity, we will need to acknowledge the privilege many of us hold and mobilise it ‘to lift people up to find solutions that work for them^65^’.
